# Measuring people’s views on health system performance: Design and development of the People’s Voice Survey

**DOI:** 10.1371/journal.pmed.1004294

**Published:** 2023-10-06

**Authors:** Todd P. Lewis, Neena R. Kapoor, Amit Aryal, Rodrigo Bazua-Lobato, Susanne Carai, Emma Clarke-Deelder, Kevin Croke, Rashmi Dayalu, Laura Espinoza-Pajuelo, Günther Fink, Patricia J. Garcia, Ezequiel Garcia-Elorrio, Theodros Getachew, Prashant Jarhyan, Munir Kassa, Soon Ae Kim, Agustina Mazzoni, Jesus Medina-Ranilla, Sailesh Mohan, Gebeyaw Molla, Mosa Moshabela, Inbarani Naidoo, Jacinta Nzinga, Juhwan Oh, Emelda A. Okiro, Dorairaj Prabhakaran, Javier Roberti, Gillian SteelFisher, Tefera Taddele, Ashenif Tadele, Xiaohui Wang, Roman Xu, Hannah H. Leslie, Margaret E. Kruk

**Affiliations:** 1 Department of Global Health and Population, Harvard T.H. Chan School of Public Health, Boston, Massachusetts, United States of America; 2 Department of Epidemiology and Public Health, Swiss Tropical and Public Health Institute, Allschwil, Switzerland; 3 University of Basel, Basel, Switzerland; 4 Division of Country Health Policies and Systems, WHO Athens Quality of Care Office World Health Organization Regional Office for Europe, Athens, Greece; 5 School of Public Health, Cayetano Heredia University, Lima, Peru; 6 Institute for Clinical Effectiveness and Health Policy, Buenos Aires, Argentina; 7 Health System & Reproductive Health Research Directorate, Ethiopian Public Health Institute, Addis Ababa, Ethiopia; 8 Public Health Foundation of India (PHFI), Gurugram, Haryana, India; 9 Minister’s Office, Ministry of Health, Addis Ababa, Ethiopia; 10 Research & Training Center, Korea Foundation for International Healthcare, Seoul, South Korea; 11 Centre for Chronic Disease Control (CCDC), New Delhi, India; 12 Deakin University, Melbourne, Victoria, Australia; 13 School of Nursing and Public Health, College of Health Sciences, University of KwaZulu-Natal, Durban, South Africa; 14 Centre for Community Based Research, Human and Social Capabilities Division, Human Sciences Research Council, Durban, South Africa; 15 Health Economics Research Unit, KEMRI-Wellcome Trust Research Programme, Nairobi, Kenya; 16 Department of Medicine, Seoul National University College of Medicine, Seoul, South Korea; 17 Population Health Unit, KEMRI-Wellcome Trust Research Programme, Nairobi, Kenya; 18 Centre for Tropical Medicine & Global Health, Nuffield Department of Medicine, University of Oxford, Oxford, United Kingdom; 19 Epidemiology and Public Health Research Centre, National Council for Scientific and Technical Research, Buenos Aires, Argentina; 20 Department of Health Policy and Management, Harvard TH Chan School of Public Health, Boston, Massachusetts, United States of America; 21 Department of Social Medicine and Health Management, School of Public Health, Lanzhou University, Lanzhou, China; 22 Department of Health Management, School of Health Management, Southern Medical University, Guangzhou, China; 23 Division of Prevention Science, University of California, San Francisco, California, United States of America

## Abstract

Todd Lewis and co-authors discuss development and use of the People’s Voice Survey for health system assessment.

**Summary points**

- People should be at the center of health system performance assessment. Populations can provide critical insight on quality of care, confidence in health services, and health outcomes.
- However, today’s measurement approaches overlook key dimensions of population perspective such as confidence in the health system. Surveys are rarely standardized to enable cross-country comparison.
- The People’s Voice Survey (PVS) aims to fill this gap. The PVS is a novel multicountry survey of people’s perspective on health system performance. It measures a wide range of domains, including health status, health system utilization patterns, ratings of care quality, and confidence and trust in the health system.
- The survey allows for a flexible, mixed mode design that uses telephone, online, and in-person data collection approaches to achieve a nationally representative sample of adults. Critically, the survey includes both health system users and non-users. It can be adapted for use in high-, middle-, and low-income countries.
- We describe the motivation for this new instrument, the multistep collaborative development and validation process, and policy use cases for 19 countries participating in the first wave of data collection.
- Findings from the survey can be used to integrate people’s voices into health system policymaking and guide strategic investments towards higher quality health systems.

## Background and introduction to the people’s voice survey

Estimates based on 2016 excess death data show that approximately 5 million people die each year in low-income and middle-income countries (LMICs) from treatable conditions despite seeking health care, pointing to a global crisis in health system quality [1]. The 2018 *Lancet Global Health* Commission on High Quality Health Systems in the Sustainable Development Goal Era (HQSS Commission) noted that large-scale health system transformations are needed to improve quality of care [2–4]. The HQSS Commission defined a high-quality health system as one that “optimizes health care in a given context by consistently delivering care that improves or maintains health outcomes, by being valued and trusted by all people, and by responding to changing population needs.” Thus, locating people at the center of health systems is of utmost importance and this requires obtaining their feedback on health system performance. This feedback can improve patient experience which is both intrinsically valuable to users and instrumentally valuable for patient safety and clinical effectiveness, including adherence to recommended clinical practice and use of preventive care [5].

There are few cross-nationally comparable instruments for tracking people’s perspectives of health system performance [6,7]. Available survey instruments, such as the Demographic and Health Surveys and Service Provision Assessments, capture a useful but limited set of concepts regarding people’s experiences of health care (Table 1). In LMICs in particular, many existing measures focus on supply-side factors (e.g., spending, provider numbers) instead of the processes and outcomes that matter most to people, such as experience of respectful care and trust in the system [8]. Further, the population’s assessment of the health system is a necessary complement to measures of health system-amenable outcomes currently in wide use [6].

**Table 1:** Select multicountry tools measuring population views on the health system in current use.

Surveys (vary by country and wave)	Aim	Population	Mode	Sample size (per country)	Key domains	No. of items	No. countries in last wave	Income levels of survey countries	Year of last wave	Organization
People’s Voice Survey	To measure people’s health care use, experiences, and overall confidence in the health system; to hold health systems accountable and enable cross-national comparisons	All adults aged 18 and older	Phone survey; face-to-face household survey in areas of low phone penetration	1,000–2,500 respondents	Health and demographics; Utilization of care and system competence; Care experience; Health system confidence	~65	19	High, middle, low	2022–2023	QuEST Network (https://questnetwork.org/peoples-voice-survey)
Service Provision Assessments	To assess service availability and quality of care within countries’ health systems at the facility level; to provide a snap-shot of the service environment, resources, and practices used to provide health services	Formal sector healthcare facilities (includes interviews and observations or providers and patient exit interviews)	In-person interviews and surveys interviewers	~400 to 700 health facilities	Inventory (resources and equipment); Health worker qualifications/skills, clinical performance, services provided, in-service training, and work environment; Client experience	~360	1 completed; 1 ongoing	Middle, low	2021	Demographic and Health Surveys Program, USAID (https://dhsprogram.com/Methodology/Survey-Types/SPA.cfm)
Demographic and Health Surveys	To provide nationally representative data on population, health, and nutrition	Households; in selected households, all women aged 15 to 49 years; may include male respondents aged 15 to 59 years	Household survey	~5,000 to 30,000 households	Demographics and health and nutrition indicators (e.g., infant and child mortality, fertility, family planning use, maternal health, child immunization, malnutrition levels, HIV prevalence, and malaria)	~467 items	1 completed 2021; 16 ongoing in 2022	Middle, low	2021–2022	Demographic and Health Surveys Program, USAID (https://dhsprogram.com/Methodology/Survey-Types/DHS.cfm)
International Health Policy Survey (2020 edition)	To compare the health experiences of adults with lower income during the pandemic and the effect of income-related disparities (e.g., health status, patient experience, barriers to care)	Noninstitutionalized adults aged 18 and older in Australia, Canada, France, Germany, the Netherlands, New Zealand, Norway, Switzerland, Sweden, the United Kingdom, and the United States	Landline and mobile phone interviews; online surveys in Sweden, Switzerland, and the United States	~607 to 4,530 participants	Health status; Socioeconomic risk factors; Affordability of care; Access to care	~130	11	High	2020 (more recent versions focused on older adults and health care providers)	Commonwealth Fund (https://www.commonwealthfund.org/publications/surveys/2020/dec/2020-international-survey-income-related-inequalities)
Consumer Assessment of Healthcare Providers and Systems (CAHPS): Clinician and Group Survey	Determine the need for improvement activities and evaluate their impact; Equip consumers with information to choose healthcare providers; Monitor the performance of providers and groups and reward them for high-quality care	Adults 18 years old or older, or children 17 years old or younger who had a visit with an individual provider, practice, or medical group	Mail, telephone, email, or mixed-mode protocols	Variable; typically requires 50 respondents per provider or 300 respondents per medical group	Patient experience of: Getting timely appointments, care, and information; How well providers communicate with patients; Providers’ use of information to coordinate patient care; Helpful, courteous, and respectful office staff; Patients’ rating of the visit/provider	33 (Adult visit survey 4.0)	Developed for the United States; implemented in 6 countries in Europe	High	N/A (Independently conducted)	United States Agency for Healthcare Research and Quality (https://www.ahrq.gov/cahps/index.html)
Primary Care Assessment Tools (PCAT), Adult and Children Editions	To assess and assure the quality of primary care service delivery	Varies; Can include children, adults, health professionals, managers, and administrators	Mail surveys and telephone interviews	Varies	Patient subjective experience measures of: Accessibility; Utilization; Ongoing care; Coordination of services; Comprehensiveness of services available and received	Varies; 74 items in the original instrument	N/A (Independently conducted)	High, middle	2021 (Brazil)	Johns Hopkins University (https://www.jhsph.edu/research/centers-and-institutes/johns-hopkins-primary-care-policy-center/pca_tools.html)
Patient-Reported Indicator Surveys (PaRIS): International Survey of People Living with Chronic Conditions	To measure the outcomes and experiences of healthcare that matter most to people	Primary care service users aged 45 years or older and providers	Online; Respondents can opt for a paper version	2,500	Subjective feelings of users about their health; Degree of decision-making involvement about their health; The extent to which chronic conditions limit participation in social activities; How outcomes and experiences vary with people from different socioeconomic background	118 (User questionnaire); 40 (Provider questionnaire)	21 (planned)	High	2023 (planned)	Organisation for Economic Co-operation and Development (OECD) (https://www.oecd.org/health/paris/)
EUROPEP (European Task Force on Patient Evaluations of General Practice)	To measure patient evaluations of specific aspects of general practice care; To enable international comparison of general practice care in Europe.	Patients recruited from general practitioner practices	Mail survey	~316–1,080	Aspects of care: Relation and communication; Medical care; Information and support; Continuity and cooperation; Facilities, availability and accessibility	23	20 (since inception)	High, middle	2021	European Society for Quality and Safety in Family Practice (https://equip.woncaeurope.org/tools/europep)

**Notes:** The table includes multicountry surveys in current use that are purpose-built for assessing health system performance. It excludes opinion polls, ad hoc surveys and event-based polling, single-country surveys, and surveys of one aspect of health system functioning, such as National Health Accounts.

Recent tools, especially in high-income countries, have begun to include a limited set of questions on these topics, though these surveys focus exclusively on recent users (e.g., patient surveys) or specific disease groups (e.g., noncommunicable diseases). Many patient experience surveys rely on in-person interviews that are expensive and cumbersome to implement [9,10]. Meanwhile, people’s opinions can shift rapidly in response to political, social, economic, and population health need changes making repeat assessment essential.

The People’s Voice Survey (PVS) is a new tool designed by the Quality Evidence for Health System Transformation (QuEST) Network, an initiative focused on measuring and improving health system quality through multicountry partnerships. The PVS is a rapid population-representative survey that aims to inform action toward more effective and people-centered health systems and promote health system accountability to populations. It can assess public sentiment on factors like confidence and trust in the health system among the full adult population in each country, including recent users of health services as in patient experience surveys, but also past and future users. This allows policymakers to understand how well the health system is serving the entire population in addition to specific age or disease groups.

The PVS was collaboratively developed by researchers in the first wave of implementing countries, policymakers, and key regional health system stakeholders. It builds upon existing tools by measuring a wider range of domains of health system performance among full populations in a rapid, cross-nationally comparable way. The findings can inform national and multinational policy decisions and evaluation of health system programs to improve health care access and quality. Through the QuEST Network, the survey methods and findings will become freely accessible to promote policy change by governments and data uptake by health system researchers.

## Survey features: Content, implementation, and validation

### Content

**Fig 1:**
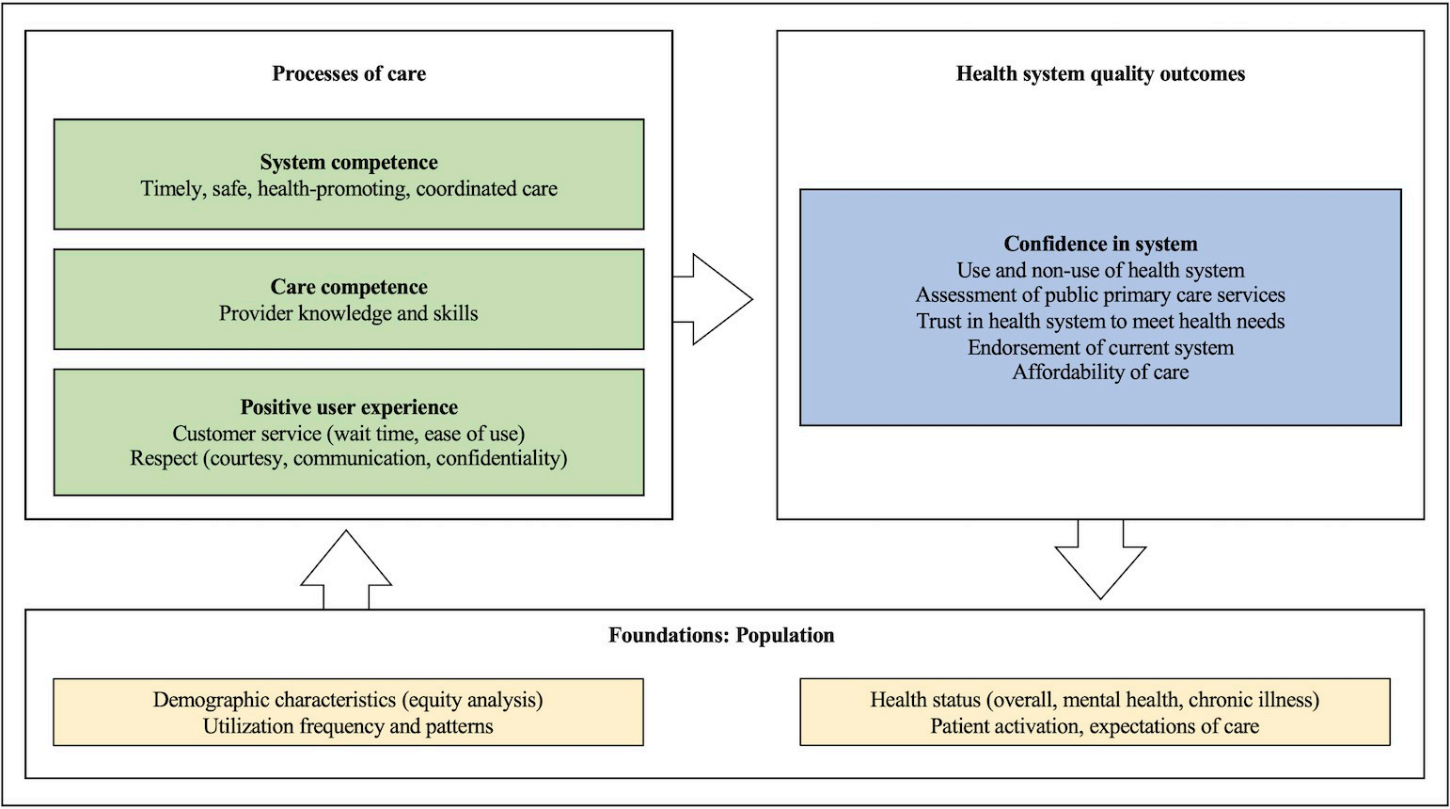
People’s Voice Survey framework. Notes: People care about outcomes beyond good health, which include trusting that the system can meet their needs, confidence that they can afford services, and endorsement of health system performance. These perceptions are informed by processes of care, including system competence (e.g., whether the health system provides coordinated, easy-to-use care integrated across platforms), care competence (e.g., provision of high-quality care from knowledgeable, high-skilled providers), and user experience (e.g., good customer service and respect). These processes and outcomes are underpinned by the foundations of the health system, including health status, demographic characteristics, patient activation, and expectations of care.

The PVS is based on the definition of a high-quality health system proposed by the HQSS Commission in 2018. The Commission emphasized that high-quality health systems need to work with people not only to improve health outcomes, but to generate trust and economic benefit for all people. The PVS focuses on the elements of system performance that are most apparent to and valued by the population, including positive user experience and confidence in the health system [2,11,12]. Fig 1 shows the main components of the PVS framework.

The PVS tool focuses on questions for which people, rather than providers, clinical observations, or facility records, are the gold standard source of information. It assesses perceptions at multiple levels of the health system, including the micro level (e.g., the point of care), the meso level (e.g., use of local facilities), and the macro level (e.g., overall health system assessment) as outlined in Box 1. Where possible, we used questions that have been validated across multiple contexts.

**Box 1. Domains of the People’s Voice Survey**

1. Health and demographics
  1.1. Demographic information
  1.2. Health status
  1.3. Patient activation
2. Utilization of care and system competence
  2.1. Usual source of healthcare
  2.2. Health service utilization patterns
  2.3. Health system competence in population health
  2.4. Non-use of healthcare
3. Care experience
  3.1. User experience and care competence
  3.2. Respondent endorsement of clinic
4. Health system confidence
  4.1. Assessment of public primary care
  4.2. Overall health system assessment
  4.3. Expectations for health system quality
5. Optional targeted modules on priority issues (e.g., COVID-19)

In addition to standard demographic questions such as age, location, and health status, we include 2 items that measure patient activation, defined as patients’ willingness and ability to take independent actions to manage their health and care, to understand people’s autonomy and decision-making power [13]. To better understand health care use patterns, we ask about frequency and heterogeneity of facility visits over the last 12 months and how users rate the quality of those visits, including for telemedicine. We also include questions on perceived medical mistakes and whether the respondent did not obtain needed care.

For those who have used care in the last 12 months, we ask them to rate multiple domains of quality such as time spent with the provider and perceived provider knowledge and skills. We include both objective and subjective rankings to gauge what is acceptable to users in different contexts. We ask whether respondents would recommend the recently used facility to a friend or family member to calculate a net promoter score, an approach to assessing whether users are “promoters” or “detractors” of health care facilities [14].

The final section of the survey assesses overall confidence in the health system, including publicly provided primary care and respondent confidence in their ability to obtain and afford high-quality care if needed. Other items include assessment of the health system trajectory (i.e., is the health system getting better or worse?) and the need for reform of the system (i.e., does the health system need major or minor changes?). As user satisfaction is deeply intertwined with people’s expectations for care, we include 2 vignettes of care to help us adjust other quality ratings in the survey for local expectations and provide more accurate cross-national comparisons.

### Implementation

**Fig 2:**
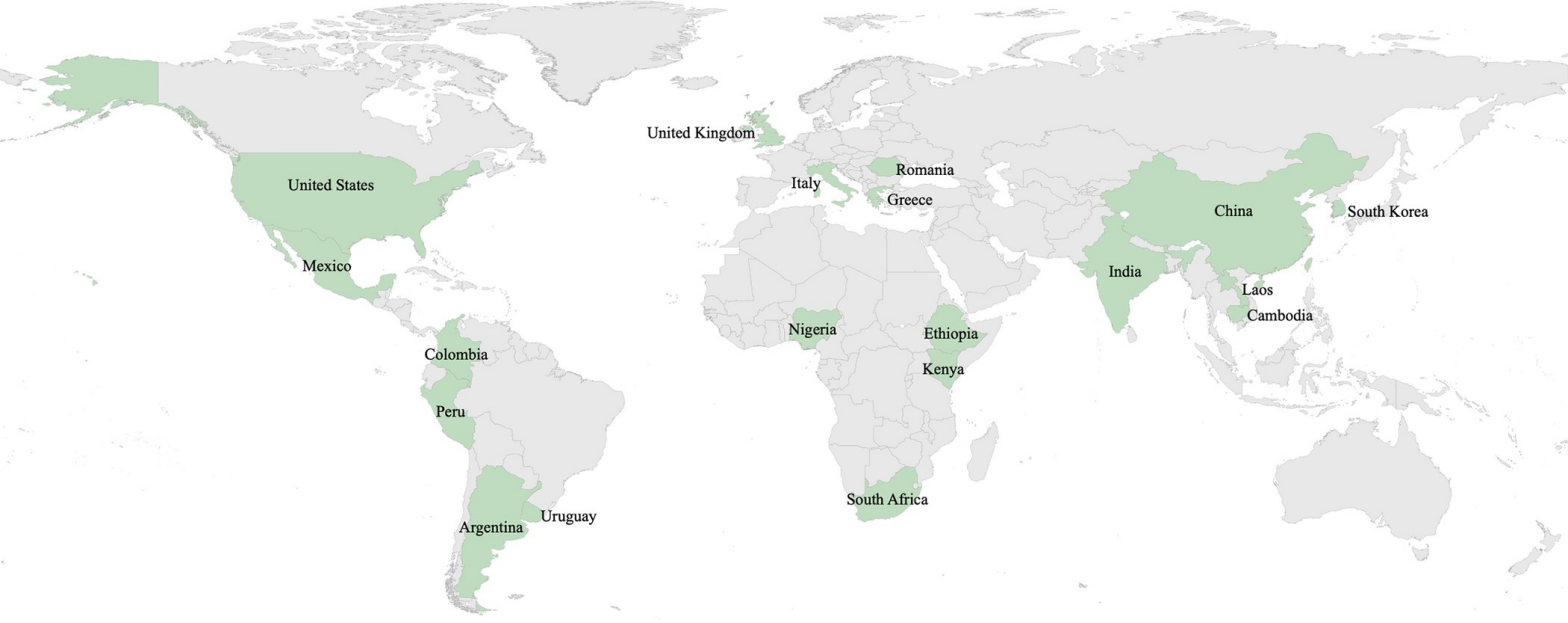
Participating countries planned for the first wave of the People’s Voice Survey. *In Argentina, the People’s Voice Survey was conducted in the Province of Mendoza only. Map shapefile available from the World Bank at: https://datacatalog.worldbank.org/search/dataset/0038272.

The PVS is designed to be used in any country regardless of region, income level, or health system structure. Implementation features are described in Table 2. The target population is all individuals aged 18 years and older; in the future, the survey could be adapted to younger individuals. The survey includes both users and non-users of the health system because health systems should be equipped to serve everyone and, especially in settings where services are supported through public funds, all people should derive value from the health system. Further, non-users may be more likely than users to have issues with the current health system and may provide insight as to how the system might best serve them as potential future consumers of health care. The Wave 1 survey has thus far included 19 countries and was available in 34 languages (Fig 2 and Table A in S1 Appendix). Details on participating countries, ethics, and funding are in Text A in S1 Appendix.

**Table 2:** Implementation features of the People’s Voice Survey.

Survey feature	Description
Survey mode	• The PVS was designed as a brief instrument to be delivered via telephone. Wave 1 implementation also included in-person and web-based delivery in some countries. Telephone-based data collection is faster and less costly than face-to-face surveys, which will promote repeated use of PVS over time. • The PVS can be delivered through other modes, including in person, online probability panels, online self-administration, and hybrid methods.
Sampling and sample size	• A minimum nationally representative sample of 1,000 respondents is recommended; a sample size of 2,000 is recommended to permit some stratified analysis. • Telephone surveys represent the adult population well in countries with minimum population telephone ownership of 80% (all but Ethiopia and Kenya among Wave 1 countries; supplemental in-person samples were added in these countries). • Random-digit dialing (RDD), known-list sampling, or sampling from an online probability panel was used. Details of this design are in Text A in S1 Appendix.
Data stewardship	• Data were cleaned and recoded in a comparable way across countries such that datasets could be easily appended for multicountry analyses and cross-national comparisons. • Code, including construction of a standard set of indicators for PVS survey items, will be available for public use. • Aggregate national data will be made available for use by policymakers, researchers, and other stakeholders as soon as available; deidentified, individual-level data will be made publicly available after a one-year embargo period following completion of data collection, analyses, and reporting by PVS collaborators.
Costs	• In Wave 1 countries, data collection took 1 to 2 months; costs per respondent ranged from $21.54 USD in India to $104.53 USD in Italy and Mexico. • Duration of data collection and costs varied by sample size, data collection partner, survey modes used, and other location-specific factors.
Collaborators	• Wave 1 countries (2022–2023) included: - Africa: Ethiopia, Kenya, Nigeria, South Africa; - Asia: Cambodia, China, India, Laos, South Korea; - Europe: Greece, Italy, Romania, United Kingdom; - North America: Mexico, United States; - South America: Argentina (Province of Mendoza only), Colombia, Peru, Uruguay. • All Wave 1 participants are research affiliates of the QuEST Network, including 2 countries identified by the World Health Organization Quality of Care and Patient Safety Office, and national partners from early stages of survey development. • The PVS is available for implementation in additional countries. All implementors must agree to shared principles for collaboration with the QuEST Network, including procedures for maintaining survey integrity and comparability as well as data sharing.

The PVS was primarily designed for telephone but is adaptable to mixed mode delivery, including in-person and web-based delivery, as required to reach the population of each country. When using telephone, we conducted interviews with the full sample of participants required in each country. Where telephone ownership was less than 80% of the population, as in Ethiopia and Kenya, we conducted an additional sample of in-person interviews to augment the full telephone sample. We also used computer-assisted web-interviewing (CAWI) when this was the most effective way to reach the population, as in the United States, United Kingdom, and South Korea.

The PVS aims to obtain population sentiment about performance of the health system by estimating population proportions agreeing with a range of statements. A survey of 1,000 individuals selected at random will produce an estimate that is within a 3% margin of error of the population proportion 95% of the time. This is the case when the prevalence is 50%; smaller numbers are needed when prevalence is higher or lower. Thus, we used a minimum sample of 1,000 in all countries. Several of our samples are larger than this to permit some stratified analysis (e.g., by urban/rural).

In each setting, detailed metadata were captured to describe the context and events surrounding data collection, such as elections, COVID-19 spikes, and major health system reforms. These data can be used to account for significant events or circumstances that may influence these cross-sectional data in dissemination efforts and to contextualize opinion data that may be perceived as unstable.

Findings will be disseminated through short fact sheets and summary briefs for policymakers and through longer survey reports that provide an in-depth look at methods and results from each survey wave. A publicly available metadata registry will contain contextual data for each wave of the survey in each country. Finally, aggregate data from surveys across countries will be available via dashboards on the QuEST Network website (www.questnetwork.org), where datasets will also be downloadable by the public.

### Development and validation

**Fig 3:**
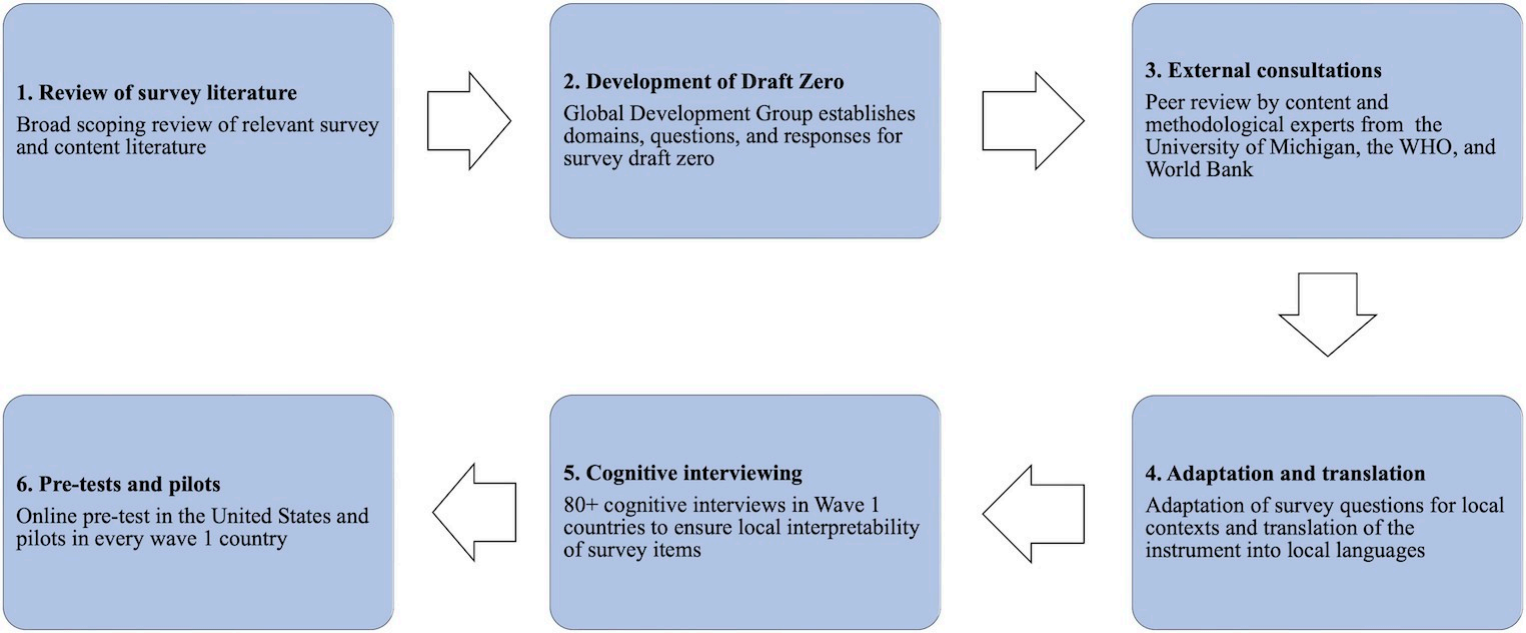
Development and validation steps of the People’s Voice Survey.

QuEST Network researchers conducted a collaborative, multistage process to develop and validate the PVS (Fig 3). To guide this process, we assembled a diverse Global Development Group (GDG) comprised of 30 health system experts from 18 high-, middle-, and low-income countries. Development group researchers conducted a broad scoping review of recent survey literature and relevant survey tools to identify questions and response options used to measure key survey domains. The GDG generated survey aims and priorities, critically appraised survey domains and items for value, clarity, and relevance, and assessed survey construction. The group met regularly over the course of 18 months in an iterative process to help ensure content validity of the survey and co-produce a “draft zero” instrument.

The GDG sought external consultation on both methodology and content of the survey. This process included peer review by survey experts from the University of Michigan and content and measurement experts from academic and multilateral organizations, including the World Health Organization (WHO) and World Bank. Reviewers assessed for best practices in survey design and delivery, such as question formulation and flow, and completeness and relevance of survey content. This feedback informed subsequent drafts and bolstered content validity of the instrument.

In Wave 1 countries, the survey instrument underwent a collaborative adaptation by local researchers and/or policymakers to ensure the instrument would be locally applicable, interpretable, and comprehensive. This helped to maintain content equivalence by ensuring cross-national comparability, clarity, and local relevance of questions in each setting. As part of this process, we conducted cognitive interviews with eligible respondents in 11 countries. Results highlighted multiple areas for improvement, including cutting lengthy items and simplifying questions with high cognitive burden.

PVS implementers in each setting identified the minimum set of survey languages necessary to reach most of the national population based on recent, nationally representative surveys. When possible, we used a team translation approach; at minimum, translation and back-translation were performed by qualified translators and closely reviewed by QuEST researchers to ensure content equivalence across languages.

To test the survey in practice, we conducted a pre-test of the full survey among a 200 person sample of respondents from an online panel in the United States. We also conducted a pilot test in one or more languages in every country. Pre-testing and pilot surveys allowed us to assess factors such as survey length, coherence, local interpretability, and quality of the response data. Additional details on steps taken to validate and adapt the PVS for participating countries are available in Text B in S1 Appendix.

## Pathways to policy

Data from the PVS will be used to answer research and policy questions to inform government practices and strengthen health system performance. Key indicators measured in the PVS are provided in Box 2. The survey will provide data that can spur health systems research, including on novel items that can inform best practices for future health system measurement, such as whether adjusting for population expectations is essential for accurately gauging health care quality ratings. PVS data are owned by collaborating researchers in the QuEST Network. As the PVS instrument and data are intended to be global public goods, they will be freely accessible to the public after a one-year embargo period to address national or regional research questions and deepen future inquiry into health systems performance.

**Box 2. Key indicators from the People’s Voice Survey**

- Health
- Health status
  ○ Mental health status
  ○ Chronic illness status
- Patient activation/empowerment
  ○ Level of activation
  ○ % of population who can bring up concerns to provider
  ○ % of population with low expectations
- Health care use
  ○ % of population with a type of insurance
  ○ % of population with usual source of care by facility ownership and level
  ○ Reasons for selecting usual source of care
  ○ Total visits, visits by facility, and type of visit (covid, virtual, home, inpatient)
  ○ Number of facilities used in past 12 months
- Care people need
  ○ Mental health service use
  ○ Respondents with chronic disease who have usual source of care
  ○ Respondents who needed but did not use health services by health status
  ○ Reasons respondents did not use care
- Health system competence
  ○ % of population who had recent screening (blood pressure, mammogram, cervical cancer, eyes, teeth, cholesterol)
- Health care quality
  ○ Quality rating of usual source of care
  ○ % of population who experienced a medical mistake in past 12 months
  ○ % of population who experienced discrimination in past 12 months
  ○ Quality of last health care visit: Overall, technical, and interpersonal quality; Service readiness; Wait time at facility; Time spent with clinician
  ○ Endorsement of usual source of care clinic
- Confidence in public primary care
  ○ Confidence in services for pregnant women, sick children, chronic illness, mental health
- Trust in the health system
  ○ Confidence in ability to get needed care
  ○ Affordability of needed care
  ○ Health system ratings (public, private, NGO)
  ○ Whether people have a say in the system
  ○ Trend in health system performance
  ○ Need for reform/health system “endorsement”
  ○ Rating of government COVID-19 management

Ideally, the PVS would be integrated as a routine component of health system planning. It can be incorporated into ongoing population surveys as an additional module or run in parallel to existing consumer surveys to provide complementary data. As a relatively low-cost, rapid survey, the PVS can be used for both ad hoc assessment at critical moments of health system evolution and longitudinal measurement of population sentiment. National governments can also use the PVS to plan for and monitor implementation of universal health coverage initiatives and inform health system design to better meet people’s needs and preferences. Multinational organizations and global partners can use the tool to monitor effectiveness of investments. Advocacy groups can measure community demand for health system improvements to better hold governments accountable. We have identified several specific policy uses for PVS in Wave 1 countries in Box 3.

**Box 3. From data to policy: Using the People’s Voice Survey for health system improvement**

1. The PVS can be embedded in routine health system evaluation to facilitate cross-national comparison and spur action by governments.
  ○ In Kenya, the PVS can be integrated into routine service readiness and provision surveys to capture the user perspective on quality.
  ○ In countries with heterogenous health system performance by state, such as India and the United States, PVS data can enable routine subnational comparisons.
  ○ The PVS can be conducted in parallel with existing consumer surveys, such as the Primary heAlth Care quAlity Cohort ChinA (ACACIA), to expand domains measured and bolster ongoing initiatives.
2. The PVS can help governments evaluate large-scale health system reforms, capturing population perspective before and after implementation.
  ○ Romania aims to improve overall quality of care and target areas such as midwifery care in light of the COVID-19 pandemic. In Greece, the government has been implementing a large-scale primary health care reform that requires patients to identify a personal doctor as a first point of care. PVS data can signal endorsement of or opposition to these changes.
  ○ In Kenya and other countries moving towards universal health coverage, the PVS can provide baseline data to motivate action and target investments.
  ○ Data can inform feasibility assessments for redesign of existing service delivery models, an approach currently underway in the Province of Mendoza, Argentina.
3. Public access to PVS data can help researchers and policymakers understand critically undermeasured areas of the health system.
  ○ Data from the PVS can be shared through regional repositories, such as the integrated African Health Observatory, and national repositories, such as the Kenya Health and Research Observatory and the Ethiopian National Data Management Center for health.
  ○ Fact sheets, summary briefs, and online dashboards can facilitate engagement with government, media, and civil society.

The PVS will be available for use by national governments, multilateral organizations, and other institutions to bring a social voice to health system planning. While the survey is designed to work in any country regardless of income-level, location, or health system structure, implementation will require (1) adaptation of a limited number of questions to the local health system context; (2) translation to local languages; (3) obtaining relevant ethical approvals where applicable; (4) establishing in-house data collection or contracting a data collection partner and tailoring data collection strategies to the national context; and (5) data sharing with PVS partners.

The QuEST Network will update and maintain the PVS based on previous survey waves and support continued expansion of the PVS network of collaborators. We aim for the survey to be an integral part of routine assessment of health system performance in multiple countries to help policymakers make smart investments that are responsive to people’s needs.

## Supporting information

S1 Appendix Table A. Implementation languages for Wave 1 of the People’s Voice Survey. Text A. People’s Voice Survey implementation. Text B. People’s Voice Survey development and validation.
(DOCX)

## Abbreviations

CAWI: computer-assisted web-interviewing
GDG: Global Development Group
LMIC: low-income and middle-income country
PVS: People’s Voice Survey
QuEST: Quality Evidence for Health System Transformation
WHO: World Health Organization
