# Supplementary material for: Measuring people’s views on health system performance: Design and development of the People’s Voice Survey
Source: PLoS Med. 2023 Oct 6;20(10):e1004294. doi: 10.1371/journal.pmed.1004294 (PMC10588880; doi:10.1371/journal.pmed.1004294)
Supplement: S1 Appendix — Implementation languages for Wave 1 of the People’s Voice Survey. Text A. People’s Voice Survey implementation. Text B. People’s Voice Survey development and validation. (DOCX) [file pmed.1004294.s001.docx]

**Appendix**

**Table A: Implementation languages for Wave 1 of the People’s Voice Survey**

| **Wave 1 country** | **Available survey languages** |
| --- | --- |
| Argentina* | Spanish |
| Cambodia | Khmer |
| China | Chinese |
| Colombia | Spanish |
| Ethiopia | Afan Oromo, Amharic, Tigrigna, Somali |
| Greece | Greek |
| India | English, Hindi, Marathi, Tamil, Telegu, Bengali, Assamese, Gujarati, Kannada |
| Italy | Italian |
| Kenya | Swahili, English |
| Laos | Lao, Khmu, Hmong |
| Mexico | Spanish |
| Nigeria | English, Hausa, Yoruba, Igbo, Pidgin |
| Peru | Spanish |
| Romania | Romanian |
| South Africa | English, Afrikaans, isiXhosa, isiZulu, Sepedi, Sesotho, Setswana |
| South Korea | Korean |
| United Kingdom | English |
| United States | English, Spanish |
| Uruguay | Spanish |

*Province of Mendoza only.

**Text A: People’s Voice Survey implementation**

*Survey mode and sampling*

In settings with lower telephone ownership (<80%), face-to-face, household-based surveys were required to fully represent the population, especially in rural areas [1,2]. In these settings, the PVS was implemented with a stratified approach that included telephone surveys in areas that had higher penetration and face-to-face surveys of people in lower-penetration areas selected through a multi-stage clustered sample design. At the first stage 15 primary sampling units (PSUs) were selected from across all rural PSUs in the countries and random walk sampling was used to select households to contact for interview. To correct for design effects, inverse probability of selection weights were constructed by country for telephone and face-to-face samples. Post-stratification weights based on external population statistics were used to adjust the sample on variables of importance to the survey, such as age, gender, location (urban/rural), and education where possible to reduce sampling biases.

Telephone surveys of patients have known limitations, such as information asymmetries between patient and provider, respondent biases, low response rates, reliance on telephone ownership, and shorter survey length which limits topics that can be covered, but may be more efficient than face-to-face surveys in generating insight on health systems [3].

*Ethical approval and funding*

In each country, ethical approval was sought according to guidelines for telephone and/or household health system assessment surveys. The PVS was funded by various sources across countries, including charitable foundations and multilateral organizations. In the future, governments could fund the survey as a national investment in routine health system assessment.

**Text B: People’s Voice Survey development and validation**

Global Development Group (GDG) members were expert in areas such as survey design, health system quality, health care preferences, patient safety, national health system organization and financing, maternal and child health, chronic diseases, mental health, primary health care, and health system policymaking. This broad range of expertise helped to ensure the content validity of the survey. The group assessed all aspects of the survey in development, including aims, methodology, and policy opportunities, with a special focus on content, including key domains overlooked in existing surveys. The GDG also investigated widely-used and validated surveys that may have been conducted in each country to understand question and response structures already in use. For example, we confirmed that Likert scale response options were in use in existing surveys in each country and used already translated scale language where possible.

Face validity of the survey was established through cognitive interviewing, which assessed whether survey items fulfilled their expected purpose (e.g., correctly understood by participants, no problems with wording, no difficulties with response options) and helped to identify any concerns about survey flow or length [4]. The GDG conducted cognitive interviews in multiple settings using a standardized, semi-structured interview guide. We used a criterion sampling approach to deliberately obtain variability by age, language, health system use, and other factors. Interviews were recorded and analyzed to identify problem areas. QuEST researchers conducted over 80 cognitive interviews in eleven countries and seven languages. Results helped to establish face validity for the instrument and highlighted multiple areas for improvement, including cutting lengthy items and simplifying questions with high cognitive burden. For example, feedback from cognitive debriefings supported the use of a single question on self-rated health instead of a multi-question scale across countries. In Colombia, Peru, and Uruguay, wording changes to questions and response options were needed to clarify types of insurance coverage and levels of care. In some countries, socio-demographic questions were adapted to local sensitivities.

We additionally conducted pretests in the United States to assess survey length and coherence. The survey was self-administered in English and Spanish to 200 respondents over age 18 years from an online panel with varying education levels and ages, as well as a small group of respondents over age 65 years who were expected to have higher health care utilization than their younger counterparts. Overall, respondents completed the survey in the expected time and found survey questions easy to answer. We made several edits based on these experiences, including removal of multiple options that were challenging for respondents.

Finally, we conducted pilot surveys in one or more languages in each country. Pilot data were used to assess concurrent validity, a form of criterion validity that examines the extent of agreement between new and previously validated measures [5]. For example, we measured the relationship between respondent education level and rating of the vignette of objectively poor quality of care and found that respondents who reported completing primary school or less were more likely to rate poor quality care as “excellent” than those who completed secondary school or higher, as found elsewhere [6]. We checked the quality of the response data, including completeness of responses, response rates, internal consistency (e.g., consistent responses on utilization patterns and number of visits), and distribution of responses (e.g., too many “I don’t know” responses). In response to the analysis of pilot data, we adjusted response options due to overuse of “other” categories, verified certain responses with external population data (e.g., COVID-19 vaccination rates), added interviewer instructions to guide respondents in areas of potential confusion, and updated relevant interviewer training materials.

**Appendix references**

1. Gourlay S, Kilic T, Martuscelli A, Wollburg P, Zezza A. Viewpoint: High-frequency phone surveys on COVID-19: Good practices, open questions. Food Policy. 2021;105: 102153. doi:10.1016/j.foodpol.2021.102153

2. Kastelic KH, Eckman S, Kastelic JG, Mcgee KR, Wild M, Yoshida N, et al. High frequency mobile phone surveys of households to assess the impacts of COVID-19 (Vol. 2): Guidelines on sampling design. 2020. Available: http://documents.worldbank.org/curated/en/742581588695955271/Guidelines-on-Sampling-Design

3. Hensen B, Mackworth-Young CRS, Simwinga M, Abdelmagid N, Banda J, Mavodza C, et al. Remote data collection for public health research in a COVID-19 era: ethical implications, challenges and opportunities. Health Policy and Planning. 2021;36: 360–368. doi:10.1093/heapol/czaa158

4. Scott K, Ummer O, LeFevre AE. The devil is in the detail: reflections on the value and application of cognitive interviewing to strengthen quantitative surveys in global health. Health Policy and Planning. 2021;36: 982–995. doi:10.1093/heapol/czab048

5. Cronbach LJ, Meehl PE. Construct validity in psychological tests. Psychological bulletin. 1955;52: 281.

6. Roder-DeWan S, Gage AD, Hirschhorn LR, Twum-Danso NAY, Liljestrand J, Asante-Shongwe K, et al. Expectations of healthcare quality: A cross-sectional study of internet users in 12 low- and middle-income countries. PLOS Medicine. 2019;16: e1002879. doi:10.1371/journal.pmed.1002879
